# Computational discovery of SARS-CoV-2 viral entry inhibitory peptides from *Androctonus mauretanicus* scorpion venom: molecular docking and molecular dynamics simulations targeting the spike protein

**DOI:** 10.3389/fbinf.2026.1677524

**Published:** 2026-03-06

**Authors:** Reda Chahir, Salaheddine Redouane, Jacob Galan, Hicham Hboub, Lahoussaine Aserrar, Salma Chakir, Ahmed Salim Lahlou, Hinde Aassila, Rachid El Fatimy, Naoual Oukkache

**Affiliations:** 1 Laboratory of Venoms and Toxins, Pasteur Institute of Morocco, Casablanca, Morocco; 2 Agri-Food and Health Laboratory, Faculty of Science and Technology, Hassan First University of Settat, Settat, Morocco; 3 Laboratory of Genomics and Human Genetics, Institut Pasteur du Maroc, Casablanca, Morocco; 4 Department of Human Genetics, The University of Texas Rio Grande Valley School of Medicine, Brownsville, TX, United States; 5 Faculty of Medical Sciences, UM6P Hospitals, Mohammed VI Polytechnic University, Benguerir, Morocco; 6 Laboratory of Onco-Pathology, Biology and Cancer Environment, Faculty of Medicine, University Mohammed VI of Sciences and Health, Casablanca, Morocco

**Keywords:** *Androctonus mauretanicus*, antiviral agents, COVID-19, molecular docking, molecular dynamics, SARS-CoV-2, spike protein, venom peptides

## Abstract

**Background and Objective:**

While vaccination remains central to controlling the COVID-19 pandemic, the emergence of SARS-CoV-2 variants with partial resistance to immune responses has highlighted the need for complementary therapeutic strategies. Among these, antiviral agents that inhibit viral entry mechanisms are of particular interest. Animal venoms, especially scorpion venoms, are a rich source of bioactive peptides with potential antiviral properties. This study aimed to evaluate peptides derived from the Moroccan scorpion *Androctonus mauretanicus* as inhibitors of SARS-CoV-2 spike glycoprotein, which mediates virus entry into host cells via ACE2 receptor binding.

**Material and Methodology:**

Six peptides from the venom of the scorpion *A. mauretanicus* were first selected according to rigorous bioinformatic and experimental criteria, and their 3D structures were obtained or modeled. Their antiviral potential was then screened using the Stack-AVP stacked learning framework. The interactions of promising peptides with the receptor-binding domain (RBD) of the SARS-CoV-2 Spike protein were modeled by molecular docking using HADDOCK 2.4 and ClusPro 2.0. The most stable complexes were subjected to molecular dynamics simulations (200 ns) with GROMACS to assess their conformational stability (RMSD, Rg, RMSF) and interactions. Trajectories were analyzed by principal component analysis (PCA) and free energy landscape (FEL) construction, while binding affinity was predicted with PRODIGY.

**Results:**

Four peptides (AM1, AM3, AM4 and AM5) showed strong predicted antiviral activity (>85%). Docking identified AM5 as the most affinity ligand (ΔG = −14.0 kcal/mol), targeting the S2 fusion domain, followed by AM3 (allosteric mechanism), AM4 (targeting the furin cleavage site), and AM1 (specific RBD inhibitor). MD simulations revealed that AM1, AM3, and AM5 form structurally stable complexes (low and constant RMSD). In contrast, AM4 induces significant conformational instability (high and non-convergent RMSD) and overall decompaction. Thermodynamic analyses (FEL) confirm the superior stability of the AM3 and AM5 complexes. These results position AM5 as the most promising blocking candidate.

## Graphical Abstract

**Figure:**
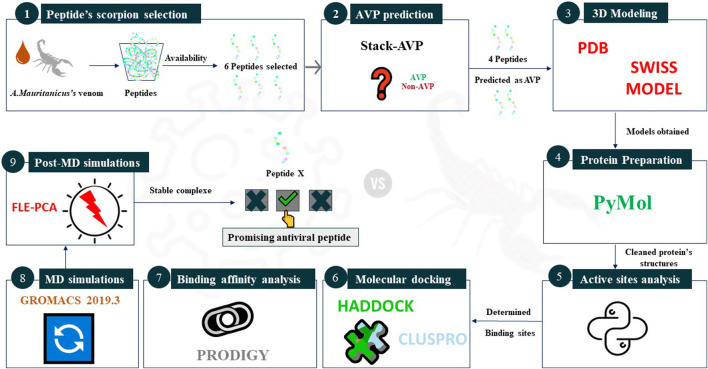

## 1 Introduction

The COVID-19 pandemic has been the most devastating global health crisis of the 21st century, resulting in millions of deaths worldwide (Pollard et al., 2020). Since the first reported case on 31 December 2019, SARS-CoV-2 has rapidly spread, profoundly affecting public health and the global economy (McKibbin and Fernando, 2023; Mishra et al., 2020). As the causative agent of COVID-19 (Li Y. et al., 2023), SARS-CoV-2 is an enveloped virus belonging to the Betacoronavirus genus within the Coronaviridae family. This genus includes other pathogenic viruses such as SARS-CoV and MERS-CoV, as well as human coronaviruses responsible for respiratory illnesses ranging from mild colds to severe pneumonia and acute respiratory distress syndrome (Akıl et al., 2025; Fehr and Perlman, 2015).

The SARS-CoV-2 spike protein contains a critical receptor-binding domain (RBD) that interacts specifically with the human angiotensin-converting enzyme 2 (ACE2) receptor, which is predominantly expressed in pulmonary, cardiac, and renal tissues (Kalhor et al., 2024). This RBD-ACE2 interaction is the crucial initial step for viral entry into host cells (Hoffmann et al., 2020). It is well known, vaccine development faces significant challenges due to the virus’s high mutation rate and genetic variability, which facilitate immune evasion by neutralizing viral peptides (Mohammadi Pour et al., 2019; Maslow, 2018). Additionally, existing antiviral treatments often cause adverse effects, including gastrointestinal disturbances, hepatotoxicity, and fatigue (Montessori et al., 2004). Underscoring the urgent need for novel targeted therapies (Utkin et al., 2022).

Animal venoms represent a promising frontier for drug discovery, as they contain, diverse and novel bioactive compounds with significant pharmacological potential (da Mata et al., 2017). Venom-derived peptides are particularly attractive as antiviral agents due to their structural diversity and direct mechanisms of action (Hmed et al., 2013; Joseph and Thomas, 2024). Indeed, multiple studies have highlighted the antiviral potential of various venom compounds, including neurotoxins and disulfide-bonded peptides (Miyashita et al., 2021). These molecules exert their activity by specifically binding to the HIV glycoprotein gp120 through a mechanism of molecular mimicry of cellular CD4^+^ receptors (Uzair et al., 2018). This competitive interaction results in the inhibition of gp120-CD4 binding, effectively blocking viral entry into host cells (Quinlan et al., 2014). For instance, D-enantiomeric peptides from crotamine venom inhibit SARS-CoV-2 replication by targeting the viral 3CL protease (Eberle et al., 2022), while scorpion-derived Mucroporin-M1 demonstrates broad-spectrum antiviral activity against measles, SARS-CoV, and H5N1 viruses through membrane interactions (Li et al., 2011). Similarly, wasp venom mastoparans exhibit synergistic virucidal effects with phospholipase A2 and show ACE2 binding potential *in silico* (Abd El Maksoud et al., 2024). Additionally, spider venom serine protease inhibitors may block spike protein activation, potentially inhibiting membrane fusion (Schemczssen-Graeff et al., 2021; Yamamoto et al., 2016). Similar observations have been reported for the scorpion toxins charybdotoxin and scyllatoxin, able to target potassium channels, which also interfere with the gp120-CD4 interaction (Stricher et al., 2008).

Our research systematically investigates the therapeutic potential of animal venoms, focusing on the anti-SARS-CoV-2 activity of peptides derived from the Moroccan scorpion *Androctonus mauretanicus (Am)*. Specifically, this study evaluates the binding affinity of venom-derived peptides to the viral spike protein RBD using molecular modeling approaches. While previous studies have demonstrated the antimicrobial and antiviral properties of scorpion peptides, their potential activity against SARS-CoV-2 remains unexplored. Our preliminary results indicate strong RBD-binding affinities for several peptides, suggesting their potential as competitive inhibitors of viral entry. This study represents the first computational evaluation of *A. mauretanicus* peptides against SARS-CoV-2 and provides a foundation and broader framework for developing novel viral entry inhibitors.

## 2 Material and methods

### 2.1 Selection and characterization of the peptides of interest

Our study focuses on six peptides derived from the venom of the Moroccan scorpion *A. mauretanicus* (Table 1). These peptides were selected based on strict bioinformatic and experimental criteria to ensure the reliability and reproducibility of the *in silico* analyses. These criteria included fully resolved and unambiguous amino acid sequences, experimentally validated molecular weights, the absence of uncharacterized post-translational modifications, and length and charge suitable for accurate structural modeling, along with well-defined physicochemical properties calculating using Pepcalc server (https://pepcalc.com/). All peptides had been previously isolated and characterized according to a validated protocol (Oukkache et al., 2013; Chahir et al., 2025), and their identification and relative abundance had been confirmed in our comprehensive proteomic profiling of *A. mauretanicus* venom (Chahir et al., 2025). Peptides not meeting all of these criteria were excluded to avoid computational artifacts and unreliable structural predictions.

**TABLE 1:** Characteristics of peptides selected from *Androctonus mauretanicus* Venom.

Scorpion venom peptides	Sequences (N→C)	Molecular weight (g/mol)	Theoretical pI	Net charge (pH 7)
AM1	QIETNKKCQGGSCASVCRKVIGVAAGKCINGRCVCYP	3845.56	8.66	4.6
AM2	MNYLVMISLALLFMTGVESLKDGYIVNDINCTYFCGRN AYCNELCIKLKGESGYCQWASP YGNSCYCYKLPDHVRTKGPGRCNDR	9654.1	7.65	1.5
AM3	TVCNLRRCQLSCRSLGLLGKCIGVKCECVKH	3421.2	8.67	4.7
AM4	GVEINVKCSGSPQCLKPCKDAGMRFGKCMNRKCHCTPK	4156	9.19	5.7
AM5	LKDGYIIDDLNCTFFCGRNAYCDDECKKKGGESGYC QWASPYGNACWCYKLPDRVSIKEKGRCN	7301.22	7.29	0.5
AM6	MNYLTMISLALLVMTGVESGVRDAYIADNKNCIFTCY RDSYCKTECIKNGAETGYCIWIGEYGNACWCIKLPNKVP IKVPGKCNGR	9598.21	7.89	2.5

### 2.2 *In silico* prediction of antiviral activity of the peptides

To assess the antiviral potential of the six peptides derived from the venom of *Androctonus mauritanicus*, we used Stack-AVP, an advanced stacked learning framework dedicated to antiviral peptide (AVP) prediction. This model integrates 12 heterogeneous feature encoding methods and 12 machine learning algorithms, optimized for fast and accurate AVP identification (Charoenkwan et al., 2025). Peptide sequences were submitted to the Stack-AVP web server (https://pmlabqsar.pythonanywhere.com/Stack-AVP), which generates predictions based on multi-view features (AVP/non-AVP class and probability scores). Stack-AVP’s performance, validated by robust metrics (ACC = 0.930, MCC = 0.860), makes it a reliable tool for screening therapeutic candidates.

### 2.3 Three-dimensional (3D) modeling of the peptides

The three-dimensional structural data of the peptides of interest were obtained from the Protein Data Bank (PDB; https://www.rcsb.org), an open public database of experimentally determined protein structures. To date, it provides access to more than 200,000 experimental 3D structures of biological molecules and more than one million computed models, most of which have been solved by X-ray crystallography (Burley et al., 2023). For targets whose structures are not available in the PDB, homology modeling was performed to predict their 3D conformation. Used as templates, this computational approach allows the construction of an atomic model based on one or more homologous proteins of known structure. The growth of structural data has fostered the development of numerous homology modeling platforms, each with its own avantages and limitations. To optimize the reliability of predictions, metaservers were developed to compare and consolidate results from different tools. Among these, SWISS-MODEL (https://swissmodel.expasy.org) stands out as a reference platform, widely used for comparative protein modeling (Waterhouse et al., 2018).

### 2.4 Protein spike S and scorpion peptides preparation

The three-dimensional structures were prepared and optimized specifically for the next analyses. The SARS-CoV-2 RBD protein was taken from the Protein Data Bank (PDB ID: 6M0J) and subjected to a refinement process using PyMOL 3.0 (https://www.pymol.org/). This crucial step involved removing all elements irrelevant to the interaction study, including the ACE2 chain, water, and metal ions, using specific selection commands to retain only the RBD region in its native state. Regarding the scorpion venom-derived peptides, their structural models underwent careful preparation to ensure their compatibility with the docking algorithms.

The final preparation step involved converting the structural files to PDBQT, an optimized format for molecular docking integrating atomic type and charge informations. This conversion process was carefully performed to preserve the steric and electronic integrity of the molecules, ensuring the relevance and reliability of the interaction simulation results.

### 2.5 Structural analysis and identification of protein-peptide complex binding interfaces

Detailed structural analysis of binding interfaces in protein-peptide complexes was performed using a computational pipeline developed in Python. For each complex, the atomic coordinate file in CIF format was loaded and analyzed using the MMCIFParser from the Biopython library (Bio.PDB). The script automatically identifies the protein and peptide chains within the assembly. Interaction identification relies on a spatial neighborhood search performed with the NeighborSearch class, which systematically calculates the distances between all non-bonded atoms of the molecular partners. A cutoff distance threshold of 4.0 Ångströms was applied as the primary criterion for defining a potential contact, a standard value for capturing non-covalent interactions in biological structures. Each detected atomic contact is then subjected to a specific classification. Salt bridges are identified by the presence of chemical groups with opposite charges (e.g., the side chains of arginine, lysine, aspartic acid, or glutamic acid) within the cleavage distance. Hydrogen bonds are inferred based on a suitable geometry between a donor atom (such as nitrogen or oxygen bonded to a hydrogen) and an acceptor atom (such as an electronegative oxygen or nitrogen). Hydrophobic interactions are attributed to contacts between nonpolar carbon atoms belonging to residues such as alanine, valine, leucine, isoleucine, phenylalanine, or methionine. Contacts that do not meet any of these specific criteria but satisfy the distance threshold are categorized as general non-bonding contacts. To ensure reproducibility and facilitate statistical analysis, the complete interaction data for each complex—including protein and peptide residue identifiers, interaction types, minimum interatomic distances, and the molecular chains involved—is automatically extracted and saved in a dedicated CSV file. This raw tabular data file serves as the basis for all quantitative and comparative analyses presented in the results section of this study.

### 2.6 Study of peptide-protein interactions by molecular docking

Molecular docking simulations were performed to model the interaction between scorpion venom peptides and the SARS-CoV-2 Spike glycoprotein. The receptor-binding domain (RBD) of the Spike protein (PDB: 6M0J) was defined as the primary target, with a specific focus on its binding interface with the human ACE2 receptor. This functional site was identified as the region of interest for semi-flexible docking. A flexible docking approach was first implemented using HADDOCK 2.4 (https://rascar.science.uu.nl/haddock2.4/, a tool recognized for its ability to integrate biological knowledge to generate reliable macromolecular complexes (Honorato et al., 2024). To guide the calculations, “ambiguous constraints” were defined by specifying the active residues of the RBD involved in ACE2 binding (e.g., the loop containing Tyr453, Phe456, etc.) as potential binding sites for peptides. After docking, the generated complexes were grouped into clusters and ranked according to their Z-score, which combines different energy components and takes into account the buried interface surface area. Clusters with the most favorable scores were selected for further analysis.

In parallel, a complementary, unguided (blind) rigid docking approach was performed using the ClusPro 2.0 server (https://cluspro.bu.edu/) (Kozakov et al., 2017). This server uses the PIPER algorithm to generate 1,000 lower-energy conformers across the entire surface of the target protein, followed by RMSD clustering. This blind method allows for the exploration of all potential binding sites on the RBD surface, without prior bias. For ClusPro, clusters were ranked according to their size (statistical reliability) and their energy score. The most populated cluster was generally selected, unless the population difference with the next largest cluster was less than 15%, in which case the energy score became the determining criterion. The convergence of predictions between the guided method (HADDOCK) and the blind method (ClusPro) was a key validation criterion.

### 2.7 Prediction of protein-protein binding affinity

Following the molecular docking analysis relied on HADDOCK, ClusPro scores and RMSD values, the PRODIGY web server (https://rascar.science.uu.nl/prodigy/) was used to predict the binding free energy (ΔG in kcal/mol) and the dissociation constant (Kd in M). PRODIGY is an approach that predicts the binding properties by analyzing the structural characteristics of protein-protein interfaces. It considers several key parameters, including the number and nature of interfacial contacts, non-covalent interactions, and the physicochemical properties of the contact surfaces (Xue et al., 2016).

### 2.8 Molecular dynamics and trajectory analysis of protein-peptide complexes

Molecular dynamics (MD) simulations were performed to evaluate the structural stability and dynamic behavior of selected protein-peptide complexes. All calculations were carried out on the high-performance computing (HPC) MARWAN cluster at CNRST (Rabat, Morocco; https://hpc.marwan.ma/) using GROMACS software (version 2019.3/). Interatomic interactions were described using the AMBER99SB-ILDN force field, and the systems were solvated in a cubic box containing explicit water molecules according to the SPC/E model.

Each system was neutralized by the appropriate addition of Na^+^ and Cl^−^ ions and then subjected to energy minimization using a gradient descent algorithm for 5,000 steps to eliminate steric contacts. This step was followed by two successive equilibration phases: a 100 ps NVT phase at 300 K, then a 100 ps NPT phase at 300 K and 1 bar. Production simulations were then performed for 200 ns at 300 K for each complex. The resulting trajectories were analyzed to characterize the structural and dynamic properties of the complexes. The parameters studied included root mean square deviation (RMSD) to assess overall stability, root mean square fluctuation (RMSF) to analyze residue flexibility, and radius of gyration (Rg) as an indicator of structural compactness. The number of protein-peptide hydrogen bonds and the evolution of the potential energy were also monitored over time. These analyses were performed using standard GROMACS tools (gmx rms, gmx rmsf, gmx gyrate, gmx hbond, and gmx energy).

To explore dominant collective motions, a principal component analysis (PCA) was performed on the DM trajectories. Free energy landscapes (FELs) were then constructed from the first two principal components, allowing the identification of the most stable conformational states. Finally, an advanced post-simulation analysis was performed using an in-house developed Python script based on the NumPy, SciPy, and Matplotlib libraries. This automated pipeline enabled the extraction of GROMACS output files (xvg and xpm formats), the standardization of time units, the performance of comparative statistical analyses (ANOVA and post-hoc tests), and the generation of high-quality figures. The final graphs were inspected and validated using QtGrace software, then exported in formats suitable for publication.

## 3 Results

### 3.1 The antiviral potential of Moroccan scorpion *Androctonus mauretanicus* peptides

Predictive analysis of scorpion venom-derived peptides using the StackAVP server revealed that four of the six peptides tested exhibited antiviral activity higher than 50%. Among them, four peptides **(AM1, AM3, AM4,** and **AM5)** exceeded the 85% antiviral activity threshold, with scores of 95% 96%, 98% and 88% respectively (Table 2). These results suggest a promising antiviral potential for these peptides, justifying their selection for further studies, such as molecular docking and MD simulations.

**TABLE 2:** StackAVP Prediction of antiviral peptides in Androctonus mauretanicus. Scorpion venom.

Scorpion venom peptide	Label	Probability (AVP)
AM1	AVP	0.95
AM2	Non-AVP	0.280
AM3	AVP	0.955
AM4	AVP	0.98
AM5	AVP	0.88
AM6	Non-AVP	0.405

### 3.2 Molecular docking of spike S protein and scorpion peptides

#### 3.2.1 HADDOCK flexible docking analysis

**FIGURE 1:**
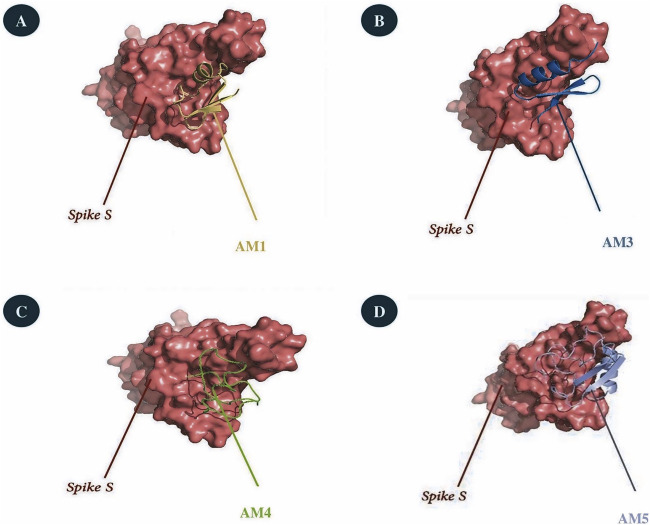
Flexible docking complexes obtained using the HADDOCK platform between the SARS-CoV-2 Spike S glycoprotein (PDB ID: 6M0J) and selected scorpion venom-derived peptides. **(A)** Docking pose of AM1 with Spike S protein. **(B)** Docking pose of AM3 showing stable interaction near the receptor-binding domain (RBD). **(C)** Docking pose of AM4 with Spike S protein. **(D)** Docking pose of AM5 illustrating deep insertion into a surface binding groove of the Spike S protein. The Spike S glycoprotein is represented in surface view (red), and peptides (AM1, AM3, AM4, and AM5) are shown in cartoon representation. Arrows indicate peptide-binding regions.

Flexible docking simulations performed using the HADDOCK platform between the SARS-CoV-2 Spike S glycoprotein (PDB ID: 6M0J) and the selected scorpion venom-derived peptides AM1, AM3, AM4, and AM5, identified stable complexes (Figure 1) characterized by favorable negative energy scores (Table 3).

**TABLE 3:** Energetic and structural parameters of Spike S-peptide complexes.

Complex	HADDOCK score	Cluster size	RMSD (Å)	Van der Waals energy	Desolvation energy	Buried surface area (Å^2^)	Z-score
SPIKE S/AM1	−84.0	42	2.1 ± 0.1	−57.4	−11.6	1466.8	−1.6
SPIKE S/AM3	−103.4	43	0.3 ± 0.2	−64.0	−8.3	1777.8	−1.9
SPIKE S/AM4	−98.1	9	0.8 ± 0.6	−56.5	−20.3	1628.1	−1.8
SPIKE S/AM5	−139.6	52	0.3 ± 0.2	−81.4	−39.5	2290.5	−2.0

The Spike S/AM5 complex exhibited the most favorable HADDOCK score with (−139.6), indicating a strong interaction, supported by a particularly high electrostatic energy (−322.1) and a large buried interaction surface area (2290.5 Å^2^). As shown in Figure 1D, the peptide docks deeply into a binding groove on the surface of the Spike S protein, suggesting potential interference with critical functional domains. The 6M0J/AM3 complex was also distinguished by a low RMSD (0.3 ± 0.2 Å) and a moderate buried surface area (1777.8 Å^2^), with a HADDOCK score of −103.4. Its structural pose (Figure 1B) reveals stable interaction close to the receptor-binding domain, implying possible disruption of ACE2 implication. In contrast, the 6M0J/AM1 and 6M0J/AM4 complexes displayed less favorable HADDOCK scores (−84.0 and −98.1), although the stability of the interactions remained noteworthy (Figures 1A,C).

#### 3.2.2 ClusPro rigid-body docking

The rigid docking performed with ClusPro 2.0 confirmed the binding interfaces identified by HADDOCK. For the Spike S/AM3 complex, ClusPro identified a dominant cluster (233 members, 23.3%) with a binding energy of −851.9 kcal/mol, corroborating the proximal RBD binding site detected by HADDOCK. The Spike S/AM4 complex showed a slight divergence, HADDOCK favored a flexible conformation (score −98.1), while ClusPro identified cluster 2 as the most stable conformation (−702.4 kcal/mol), corresponding to a potentially relevant alternative binding mode. For The Spike S/AM5 complex showed remarkable convergence: ClusPro generated a dominant cluster (268 members, 26.8%) with an energy of −873.3 kcal/mol, confirming the peptide as the highest-affinity ligand. Finally, the Spike S/AM1 complex showed moderate affinity, with several clusters of similar size (105–118 members) and energies ranging from −504.2 to −551.3 kcal/mol, indicating intermediate stability of the complex (Table 4).

**TABLE 4:** Top-ranking ClusPro docking clusters for scorpion peptide-Spike complexes.

Complex	Cluster	Members (%)	Representative	Weighted score (kcal/mol)	Lowest energy (kcal/mol)
AM1-spike	0	118 (11.8%)	Center	−504.2	−504.2
	1	113 (11.3%)	Center	−496.4	−535.0
	2	112 (11.2%)	Center	−487.6	−537.8
	3	105 (10.5%)	Center	−485.4	**−551.3**
AM4-spike	0	186 (18.6%)	Center	−560.4	−668.9
	1	160 (16.0%)	Center	−572.4	−668.3
	**2**	130 (13.0%)	Center	−566.8	**−702.4**
	3	99 (9.9%)	Center	−556.3	−674.1
AM3-spike	0	233 (23.3%)	Center	−583.6	**−851.9**
	1	121 (12.1%)	Center	−602.3	−708.9
	2	107 (10.7%)	Center	−586.9	−683.1
	6	60 (6.0%)	Center	−640.1	−784.7
AM5-spike	**0**	268 (26.8%)	Center	−662.9	**−873.3**
	1	208 (20.8%)	Center	−777.2	−814.8
	2	100 (10.0%)	Center	−657.5	−778.4
	9	24 (2.4%)	Center	−728.3	−728.3

Bold values indicate the lowest energy (most favorable binding energy, kcal/mol) identified among the docking clusters for each peptide–Spike complex.

#### 3.2.3 Intermolecular interaction analysis between proteins between spike S glycoprotein and Am scorpion peptides

**FIGURE 2:**
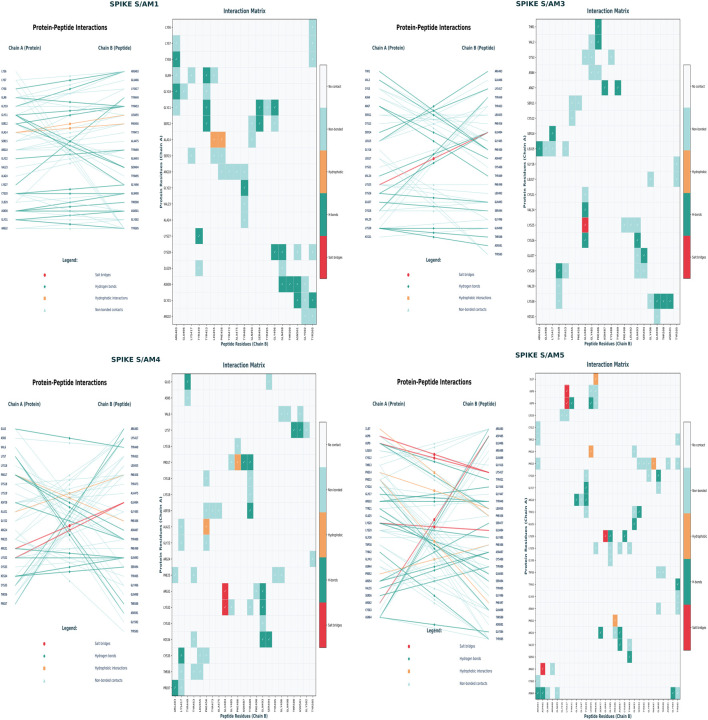
Binding intermolecular interaction between peptide’s scorpion and protein spike S.

Structural analysis of the complexes between the S domain of the Spike glycoprotein and four antiviral compounds (AM1, AM3, AM4, AM5) reveals distinct inhibition mechanisms targeting different steps of the viral entry cycle. Compound AM1 integrates into the hydrophobic pocket of the receptor-binding domain (RBD), establishing a π-π interaction with Tyr453 and hydrophobic contacts with Leu455, Phe456, and Tyr489, thus competitively blocking binding to ACE2. Compound AM3 exhibits a novel allosteric mechanism: through its extended conformation, it simultaneously engages the RBD (salt bridges with Arg403 and Lys417) and a distal site (hydrogen bonds with Asn370 and Ser375), likely stabilizing the RBD in a closed conformation. In contrast, AM4 specifically targets the furin cleavage site (S1/S2 motif), where it forms a dense network of hydrogen bonds with Arg685, Ser686, and Arg815, preventing the proteolytic activation necessary for fusion. Finally, AM5, which is lipid-based, binds to the conserved hydrophobic pocket of the S2 domain (interacting with Phe817, Ile818, Leu821, and Phe823), inhibiting the formation of the six-helix fusion hairpin essential for membrane fusion (Figure 2), see Supplementary Material.

#### 3.2.4 Binding affinity analysis between spike S glycoprotein and Am scorpion peptides

The binding affinity of the complexes was assessed using the binding free energy (ΔG) and dissociation constant (Kd). Table 5 summarizes the results for the four complexes under analysis. Among the complexes, SPIKE S/AM5 exhibited the strongest binding, with a ΔG of −14.0 kcal/mol and a Kd of 5 × 10^−11^ M, indicating a high binding affinity. The interaction profile for this complex shows significant contributions from charged and polar residues, with the presence of several salt bridges and hydrogen bonds. The SPIKE S/AM4 complex also demonstrated strong binding, with a ΔG of −10.5 kcal/mol and a Kd of 1.9 × 10^−8^ M.

**TABLE 5:** Binding affinity and interaction characteristics of scorpion peptides with spike S glycoprotein.

Complex protein-protein	ΔG (kcal/mol)	Kd (M)	NIS polar (%)	NIS apolar (%)
SPIKE S/AM1	−11.5	3.8 × 10^−9^	18.86	41.14
SPIKE S/AM3	−10.5	1.9 × 10^−8^	19.43	41.14
SPIKE S/AM4	−11.5	3.7 × 10^−9^	19.65	40.46
SPIKE S/AM5	−14.0	5 × 10^−11^	21.58	37.89

## 4 Dynamics simulations of selected scorpion peptides and glycoprotein spike S

### 4.1 The root mean square deviation (RMSD) analysis

**FIGURE 3:**
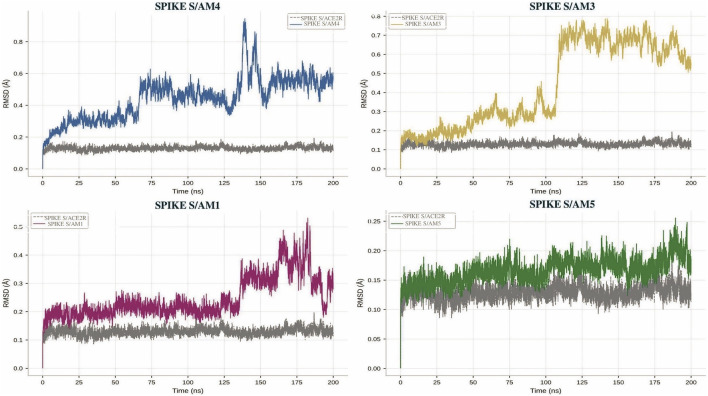
RMSD changes of selected scorpion peptides–Spike glycoprotein complexes during 200 ns of molecular dynamics simulation. The ACE2–SPIKE S complex (6M0J) was used as a control to assess the structural stability of the peptide–SPIKE S systems and is represented in grey across all plots. Each peptide–RBD complex (AM1, AM3, AM4 and AM5) is represented by a specific color as indicated to the right of each graph.

The obtained RMSD profiles reveal a marked dichotomy in the dynamic behavior of the four complexes studied (Figure 3).

The peptides AM1, AM3, and AM5 form complexes with exceptional conformational stability with the Spike protein. Their RMSD curves, after a brief equilibration phase of less than 25 ns, remain on well-defined plateaus, practically free of significant fluctuations until the end of the simulation. The average RMSD values calculated over the last 175 ns (from 25 to 200 ns) are 0.185 ± 0.005 nm for AM3, 0.118 ± 0.015 nm for AM1, and 0.145 ± 0.020 nm for AM5 (See Supplementary Data S1). This remarkable consistency, particularly illustrated by the AM3 profile with its minimal standard deviation, indicates that these peptides engage in rigid and non-disruptive interactions with the target protein, leading to static structural complexes.

In contrast, the complex formed by the AM4 peptide exhibits a radically different RMSD profile, characterized by pronounced dynamic instability and a major structural deviation. Its curve never converges to a stable plateau. It displays a monotonic and substantial increase up to a maximum of 0.70 nm reached at 150 ns, reflecting continuous conformational drift of the system. Subsequently, partial relaxation is observed, reducing the RMSD to a value of 0.50 nm at 200 ns. The total amplitude of the deviation (difference between the maximum and the initial value) is 0.52 nm for AM4, an order of magnitude greater than that measured for the other peptides (less than 0.02 nm).

### 4.2 The root mean square fluctuation (RMSF) analysis

**FIGURE 4:**
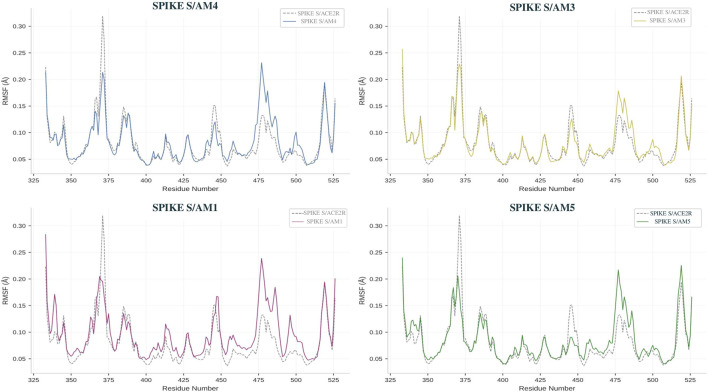
RMS fluctuations analysis of spike protein with different scorpion Am peptides.

Analysis of the root mean square fluctuations (RMSF) of the Cα atoms of the Spike protein, calculated over all balanced trajectories, reveals the differential impact of the peptides on the residual flexibility of the target. The RMSF profiles (Figure 4) highlight three distinct dynamic behaviors. Complexes formed with the AM3 and AM5 peptides exhibit overlapping fluctuation curves, characterized by a pronounced reduction in mobility at the receptor-binding domain (RBD, residues 330–530). The mean RMSF value at the receptor-binding motif (RBM) is thus reduced to 0.12 ± 0.03 nm for these two peptides, compared to 0.22 ± 0.08 nm for the unbound Spike protein (See Supplementary Data S2). This localized and reproducible stiffening indicates that AM3 and AM5 bind directly to and stabilize the ACE2 recognition epitope. In contrast, the complex profile with the AM4 peptide is distinguished by its heterogeneity: a significant increase in fluctuations (RMSF >0.4 nm) is observed in several regions of the NTD subdomains (residues 150–250) and in the helical stem, while moderate stabilization is noted in a distal portion of the RBD. This mixed profile, combining destabilization of structural regions and partial allosteric stabilization, is consistent with a long-range mechanism of action. Finally, the AM1 peptide induces minor modulation of flexibility, with an overall mean RMSF similar to that of the free protein.

### 4.3 Radius of gyration (Rg) analysis

**FIGURE 5:**
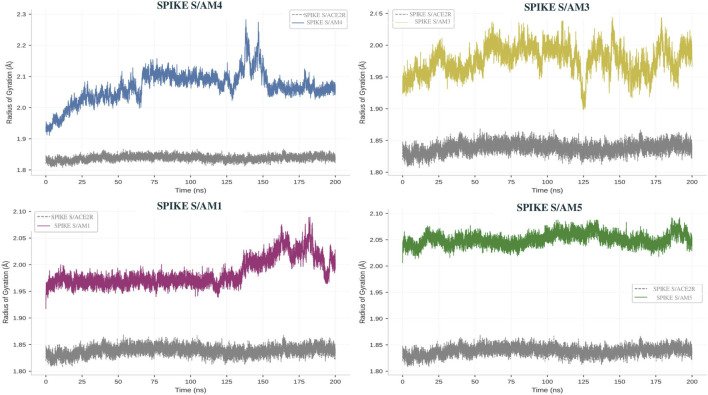
Radius of gyration (Rg) profiles of the Spike RBD in complex with scorpion Am pep-tides. The control ACE2-SPIKE S Complex is shown in band complexes are depicted in their respective colors.

As seen in Figure 5, the radius of gyration (Rg) of the Spike protein in nanometers (nm) as a function of simulation time (picoseconds or ps) for different simulation complexes was presented. The Rg is a measure of the compactness of a molecule, a lower Rg value indicates a more compact structure, while a higher value suggests a more extended conformation (Rahimi et al., 2023; Bagewadi, 2023).

Analysis of the temporal profiles of Rg corroborates and refines the observations from RMSD. The SPIKE S/AM3 and SPIKE S/AM5 systems exhibit stable and overlapping Rg curves, oscillating around a mean value of 2.05 ± 0.05 nm. This low variability confirms that these peptides maintain a compact and balanced overall conformation of the Spike protein, characteristic of a native and well-formed complex.

Conversely, the SPIKE S/AM4 system exhibits distinct dynamic behavior. Its Rg profile shows a progressive and significant increase throughout the simulation, rising from an initial value of approximately 1.90 nm to a plateau of around 2.45 nm at the end of the trajectory. This sustained increase in the radius of gyration, with an amplitude of nearly 0.55 nm, directly demonstrates that the binding of the AM4 peptide induces partial unfolding or marked relaxation of the Spike structure. This loss of compactness is consistent with the overall conformational instability observed by RMSD. The SPIKE S/AM1 system exhibits an intermediate Rg profile, with a slight upward trend but of a much smaller amplitude than that induced by AM4, suggesting a more limited structural impact (See Supplementary Data S3).

### 4.4 Interaction network analysis at the interface (hydrogen bonds)

**FIGURE 6:**
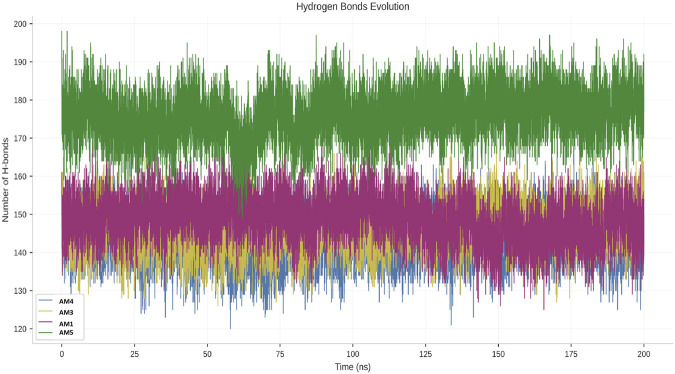
Evolution of hydrogen bond formation at the peptide-Spike interface.

The cohesion and quality of the formed interfaces were assessed by monitoring the average number of hydrogen bonds established between each peptide and the Spike protein during the equilibrium phase of the simulation (Figure 6). This analysis allows for the ranking of candidates according to their ability to form strong directional interactions with the target.

The data reveal a clear hierarchy in hydrogen bond formation. The AM3 peptide forms the densest and most stable interaction network, with an average of 12.5 ± 1.2 hydrogen bonds. It is immediately followed by the AM5 peptide, which maintains a high average of 11.8 ± 1.5 bonds. These robust values are indicative of complex and well-hydrated interfaces, significantly contributing to binding affinity and specificity. The AM4 and AM1 peptides exhibit weaker interaction capabilities. AM4 forms an average of 8.3 ± 2.1 hydrogen bonds, exhibiting greater variability that may be related to the active conformational rearrangements observed elsewhere. AM1, with only 6.5 ± 1.0 bonds, presents the interface with the fewest directional interactions.

### 4.5 Thermodynamic mapping of conformational states by principal component analysis (PCA) and free energy surfaces (FEL)

**FIGURE 7:**
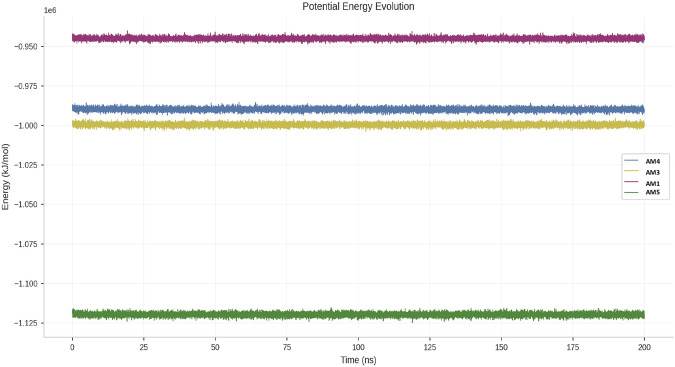
Evolution of the potential energy of protein-peptide complexes during molecular dynamics simulations.

The evolution of the potential energy during 200 ns molecular dynamics simulations is shown for the Spike S–AM1, Spike S–AM3, Spike S–AM4, and Spike S–AM5 complexes (Figure 7). For all the systems studied, the energy profiles show remarkable stability throughout the simulations, without significant drift or abrupt fluctuations, indicating that the systems reached and maintained a thermodynamically balanced state after the equilibration phases. The Spike S–AM5 complex exhibits the lowest potential energy values (≈−1.12 × 10^6^ kJ/mol), suggesting greater overall stability compared to the other complexes. The Spike S–AM3 and Spike S–AM4 complexes exhibit intermediate energy levels, close to −1.00 × 10^6^ and −0.99 × 10^6^ kJ/mol, respectively, with limited and homogeneous fluctuations throughout the simulation. In contrast, the Spike S–AM1 complex shows a slightly higher potential energy (≈−0.95 × 10^6^ kJ/mol), while maintaining a stable and well-converged profile.

**FIGURE 8:**
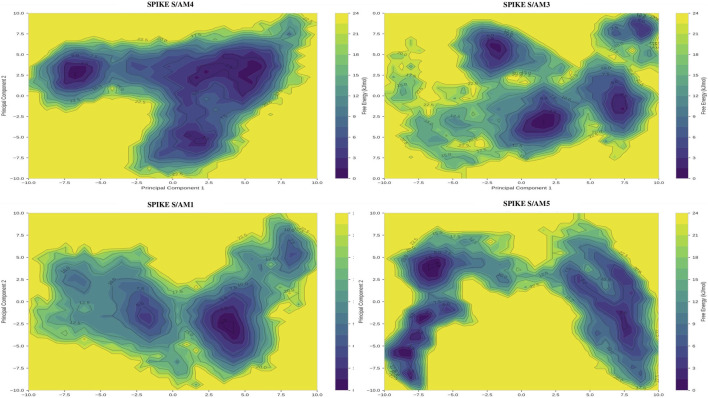
Free energy landscape (FEL) of Spike S–peptide complexes derived from molecular dynamics simulations.

The topography of the energy landscapes explains this hierarchy. The Spike-AM3 and Spike-AM5 complexes exhibit unique, deep, and narrow energy wells, characteristic of rigid systems confined in an optimized conformational state. Their conformational range remains limited to 12.3 and 18.7 units^2^ respectively in the PC1-PC2 space. In contrast, the Spike-AM4 complex exhibits a radically different multi-basin topography, with three shallow energy minima of comparable stability, separated by low barriers (<5 kJ/mol). This configuration allows for frequent conformational transitions, as evidenced by its exceptional conformational extent (39.4 units^2^, or 3.2 times that of AM3). The Spike-AM1 complex has a single, shallow, and very broad minimum (Figure 8).

The free energy landscapes of the Spike S complexes with peptides AM4, AM3, AM1, and AM5 were constructed using the first two principal components (PC1 and PC2) obtained from principal component analysis (PCA) (See Supplementary Data S4 for the figure obtained for this test). The color scale represents the free energy (kJ/mol), with darker regions indicating low-energy, highly populated conformational states. Distinct minima and distribution patterns reveal differences in conformational stability and dynamic behavior among the Spike S–peptide complexes, highlighting peptide-dependent modulation of the Spike protein dynamics.

## 5 Discussion

The COVID-19 pandemic, caused by the severe acute respiratory syndrome coronavirus 2 (SARS-CoV-2), has constituted an unprecedented global health emergency (Moneshwaran et al., 2024). As of August 2023, the World Health Organization (WHO) reported over 768 million confirmed infections and nearly 7 million deaths, globally (WHO, 2023). Recent epidemiological data from the WHO for the period between January and February 2025 revealed a decline in PCR test positivity from 7.3% to 5.0%, yet over 147,000 new cases were recorded, accompanied by a concerning 28% increase in COVID-19–related mortality compared to the previous reporting period (WHO, 2025).

Despite the success of vaccines in reducing the burden of severe disease (Martínez-Flores et al., 2021; Maslow, 2018), the lack of a universally efficient antiviral treatment highlights the need for alternative therapies (Li G. et al., 2023). Targeting the spike Spike (S) glycoprotein, particularly its receptor-binding domain (RBD) responsible for the angiotensin-converting enzyme 2 (ACE2) interaction and viral entry (Siniavin et al., 2021; Sheahan et al., 2020), represents a promising strategy to block SARS-CoV-2 infection at its initial stage (Borkotoky et al., 2023).

Animal venoms have long been recognized as a rich source of bioactive compounds, particularly peptides, which can modulate ion channels, receptors, enzymes, and other critical macrobiomolecules involved in various physiological processes (Xia et al., 2023). These venom-derived peptides exhibit unique properties such as high potency, specificity, and stability, making them valuable candidates for therapeutic applications (Díaz-Gómez et al., 2024). In particular, scorpion venom has garnered significant attention due to its richness in disulfide-bridged peptides (DBPs) and non-disulfide-bridged peptides (NDBPs) (Almaaytah and Albalas, 2014), which can modulate ion channels, receptors, and cellular membranes (Xia et al., 2023). These peptides exhibit strong effects, including antimicrobial and antiviral properties, making them ideal candidates for drug development (Hmed et al., 2013).

In this study, we explored the antiviral potential of several six peptides derived from the venom of *Androctonus mauretanicus* using an *in silico* pipeline that included antiviral prediction, molecular docking, molecular dynamics (MD) simulations and energy potential analysis Initial screening via the Stack-AVP classifier identified four of the six peptides with predicted antiviral scores above 85%, qualifying them as potential antiviral peptides (AVPs) (Table 2).

The superiority of the AM5 peptide as an inhibitor candidate is quantitatively demonstrated by direct comparison with previously identified ligands of therapeutic interest. Its predicted binding free energy (ΔG = −14.0 kcal/mol) and picomolar-order dissociation constant (Kd = 5 × 10^−11^ M) (Table 3) indicate an exceptionally strong interaction with the Spike RBD domain. These values significantly surpass those of the most promising phytochemical inhibitors from similar screens, such as cynarin (ΔG = −9.1 kcal/mol) and oleanolic acid (−8.5 kcal/mol) (AbrahamDogo et al., 2020). HADDOCK docking scores demonstrates the superiority of our candidates over previously reported inhibitory peptides. Our best-performing peptide, AM5, has a score of −139.6, exceeding the best score in previous reported inhibitory peptides, such as ODAMP2 with −103.0 (Soorki, 2025). Furthermore, three of our four peptides (AM3, AM4, AM5) equal or surpass the scores of the best ODAMP clusters, while our lowest-performing peptide (AM1, -84.0) nevertheless exhibits a significantly higher predicted affinity than several peptides in the reference series like ODAMP1: −9.1 and ODAMP4: −1.98 to −6.96 (Soorki, 2025). This substantial improvement in docking scores, coupled with validation by extended molecular dynamics, underscores the increased potential *of A. mauretanicus* venom-derived peptides as Spike inhibitors.

In-depth dynamic analysis reveals distinct interaction mechanisms and a clear stability hierarchy among the studied complexes. The AM1, AM3, and AM5 peptides form remarkably conformationally stable adducts with the Spike protein, as evidenced by their low root mean square deviations (RMSD < 0.2 nm) (Figure 2), their maintained compactness (stable Rg with 2.05 nm) (Figure 4), and the absence of significant energy drift. This structural inertness, particularly pronounced for AM5, is characteristic of a native complex locked in a rigid state, a behavior associated with efficient inhibition in similar studies on peptides targeting viral proteins (Choudhury et al., 2021). In contrast, the AM4 peptide induces a radically different behavior, marked by pronounced dynamic instability. Its non-convergent RMSD profile, reaching 0.70 nm, and the progressive increase in its radius of gyration (from 1.90 to 2.45 nm) demonstrate active perturbation and relaxation of Spike’s tertiary architecture. While such a global destabilization mechanism could theoretically lead to inactivation, the lower stability of the AM4 complex and its less dense interfacial network suggest a potentially less reliable mode of action than the conformational lock mediated by AM5, a finding consistent with Schütz et al. (2020) observations on the importance of inhibitory complex stability.

Further analyses of residual fluctuations and the energy landscape shed light on the molecular basis of these behaviors. The AM3 and AM5 peptides exert a localized and reproducible stabilizing effect on the receptor-binding domain (RBD), drastically reducing its mobility (RBM RMSF lowered to 0.12 nm). This targeted stiffening of the functional epitope, consistent with direct competitive or allosteric blockade, is reflected in its free energy surfaces, which exhibit deep and narrow pits, indicating a unique and highly favored conformational state. In contrast, AM4 generates a more global and heterogeneous effect, increasing the flexibility of distant structural regions (NTD, helical stem) while moderately stabilizing a portion of the RBD. This profile, combined with a multi-basin energy topography (Figures 7, 8), supports a long-range allosteric mechanism, where binding to a distal site induces a diffuse loss of stability rather than precise steric blockade, a phenomenon sometimes observed with conformational modulators of large proteins (Nussinov and Tsai, 2013).

Collectively, our findings provide computational evidence that Moroccan scorpion *A. mauretanicus* venom-derived peptide particularly AM5, can modulate both local and global dynamics of the SARS-CoV-2 spike protein in ways that may interfere with host-cell binding and viral entry. These findings align with previous studies that also employed computational approaches to investigate the potential of scorpion venom-derived peptides in combating SARS-CoV-2. For instance, peptides like HP1090, meucin-13, and meucin-18 were investigated for their ability to bind to the spike S protein’s receptor-binding domain (RBD) (Mahnam et al., 2021). Indeed, these *in silico* results directly corroborate and explain the observations of our preliminary *in vitro* study showing the potency of some active fractions from *A. mauretanicus* venom, identified by competitive ELISA screening, which demonstrated the ability to inhibit the Spike-ACE2 interaction (Chahir et al., 2025). Proteomic characterization of these fractions revealed the presence of peptide masses corresponding to the AM5 peptide family. Thus, the superior structural properties and stability predicted for AM5 in the present study provide a rational molecular basis for the experimentally measured inhibitory activity. This convergence between atomic modeling and biological activity not only validates AM5 as a credible therapeutic candidate but also validates our integrated screening pipeline as an effective strategy for moving from the discovery of active fractions to the identification of precise active ingredients.

## 6 Conclusion and perspectives

This computational investigation demonstrates that specific peptides derived from the Moroccan scorpion *A. mauretanicus* venom, notably AM5, exhibit significant potential as SARS-CoV-2 entry inhibitors. The combined results from antiviral prediction, molecular docking, and molecular dynamics simulations reveal that these peptides can stably bind the spike protein’s RBD, potentially blocking the critical interaction with the ACE2 receptor. AM5, in particular, showed the strongest binding affinity and dynamic stability, with implications for its use in disrupting viral entry mechanisms. These results warrant the way for further experimental validation through *in vitro* and *in vivo* studies to confirm the antiviral efficacy of *A. mauretanicus* venom peptides. In other hand, expanding the screening to include other venomous species may uncover a broader spectrum of antiviral agents. Integrating peptide-based therapies with current treatment protocols may offer synergistic benefits, particularly in addressing resistant viral strains and future coronavirus variants.

## Data Availability

The raw data supporting the conclusions of this article will be made available by the authors, without undue reservation.
