# Correction: Computational discovery of SARS-CoV-2 viral entry inhibitory peptides from *Androctonus mauretanicus* scorpion venom: molecular docking and molecular dynamics simulations targeting the spike protein

**DOI:** 10.3389/fbinf.2026.1826409

**Published:** 2026-03-19

**Authors:** Reda Chahir, Salaheddine Redouane, Jacob Galan, Hicham Hboub, Lahoussaine Aserrar, Salma Chakir, Ahmed Salim Lahlou, Hinde Aassila, Rachid El Fatimy, Naoual Oukkache

**Affiliations:** 1 Laboratory of Venoms and Toxins, Pasteur Institute of Morocco, Casablanca, Morocco; 2 Agri-Food and Health Laboratory, Faculty of Science and Technology, Hassan First University of Settat, Settat, Morocco; 3 Laboratory of Genomics and Human Genetics, Institut Pasteur du Maroc, Casablanca, Morocco; 4 Department of Human Genetics, The University of Texas Rio Grande Valley School of Medicine, Brownsville, TX, United States; 5 Faculty of Medical Sciences, UM6P Hospitals, Mohammed VI Polytechnic University, Benguerir, Morocco; 6 Laboratory of Onco-Pathology, Biology and Cancer Environment, Faculty of Medicine, University Mohammed VI of Sciences and Health, Casablanca, Morocco

**Keywords:** *Androctonus mauretanicus*, antiviral agents, COVID-19, molecular docking, molecular dynamics, SARS-CoV-2, spike protein, venom peptides

Affiliation 5 Faculty of Medical Sciences, UM6P Hospitals, Mohammed VI Polytechnic University, Benguerir, Morocco was erroneously given as Institute of Biological Sciences (ISSB), Faculty of Medical Sciences (FMS), Mohammed VI Polytechnic University (UM6P), Ben Guerir, Morocco.

Affiliation 6 Laboratory of Onco-Pathology, Biology and Cancer Environment, Faculty of Medicine, University Mohammed VI of Sciences and Health, Casablanca, Morocco was erroneously given as Faculty of Medical Sciences, UM6P Hospitals, Mohammed VI Polytechnic University, Benguerir, Morocco.

The author contributions statement was erroneously given as “RC: Conceptualization, Data curation, Formal Analysis, Investigation, Methodology, Software, Supervision, Visualization, Writing–original draft, Writing–review and editing. SR: Conceptualization, Data curation, Formal Analysis, Investigation, Methodology, Software, Supervision, Validation, Visualization, Writing–review and editing. JG: Formal Analysis, Supervision, Validation, Visualization, Writing–review and editing. HH: Conceptualization, Formal Analysis, Software, Visualization, Writing–review and editing. LA: Conceptualization, Data curation, Formal Analysis, Investigation, Methodology, Software, Validation, Visualization, Writing–review and editing. SC: Data curation, Investigation, Writing–review and editing. AL: Data curation, Investigation, Visualization, Writing–review and editing. HA: Investigation, Supervision, Validation, Visualization, Writing–review and editing. RE: Conceptualization, Formal Analysis, Funding acquisition, Methodology, Project administration, Resources, Software, Validation, Visualization, Writing–review and editing. NO: Conceptualization, Data curation, Formal Analysis, Funding acquisition, Investigation, Methodology, Project administration, Resources, Software, Supervision, Validation, Visualization, Writing–review and editing.” The correct author contributions statement is:

“RC: Conceptualization, Data curation, Formal analysis, Investigation, Methodology, Software, Visualization, Writing–original draft, Writing–review and editing. SR: Conceptualization, Data curation, Formal analysis, Investigation, Methodology, Software, Validation, Visualization, Writing–review and editing. JG: Formal analysis, Validation, Writing–review and editing. HH: Formal analysis, Software, Writing–review and editing. LA: Conceptualization, Data curation, Formal analysis, Investigation, Methodology, Software, Validation, Visualization, Writing–review and editing. SC: Data curation, Investigation, Writing–review and editing. AL: Data curation, Investigation, Visualization, Writing–review and editing. HA: Investigation, Validation, Supervision, Writing–review and editing. RE: Conceptualization, Funding acquisition, Project administration, Resources, Validation, Writing–review and editing. NO: Conceptualization, Data curation, Formal Analysis, Funding acquisition, Investigation, Methodology, Project administration, Resources, Software, Supervision, Validation, Visualization, Writing–review and editing.”

The original article has been updated.

