# Supplementary material for: Computational discovery of SARS-CoV-2 viral entry inhibitory peptides from Androctonus mauretanicus scorpion venom: molecular docking and molecular dynamics simulations targeting the spike protein
Source: Front Bioinform. 2026 Mar 6;6:1677524. doi: 10.3389/fbinf.2026.1677524 (PMC13044517; doi:10.3389/fbinf.2026.1677524)
Supplement: Supplementary file 1 [file DataSheet1.docx]

**SUPPLEMENTARY DATA**


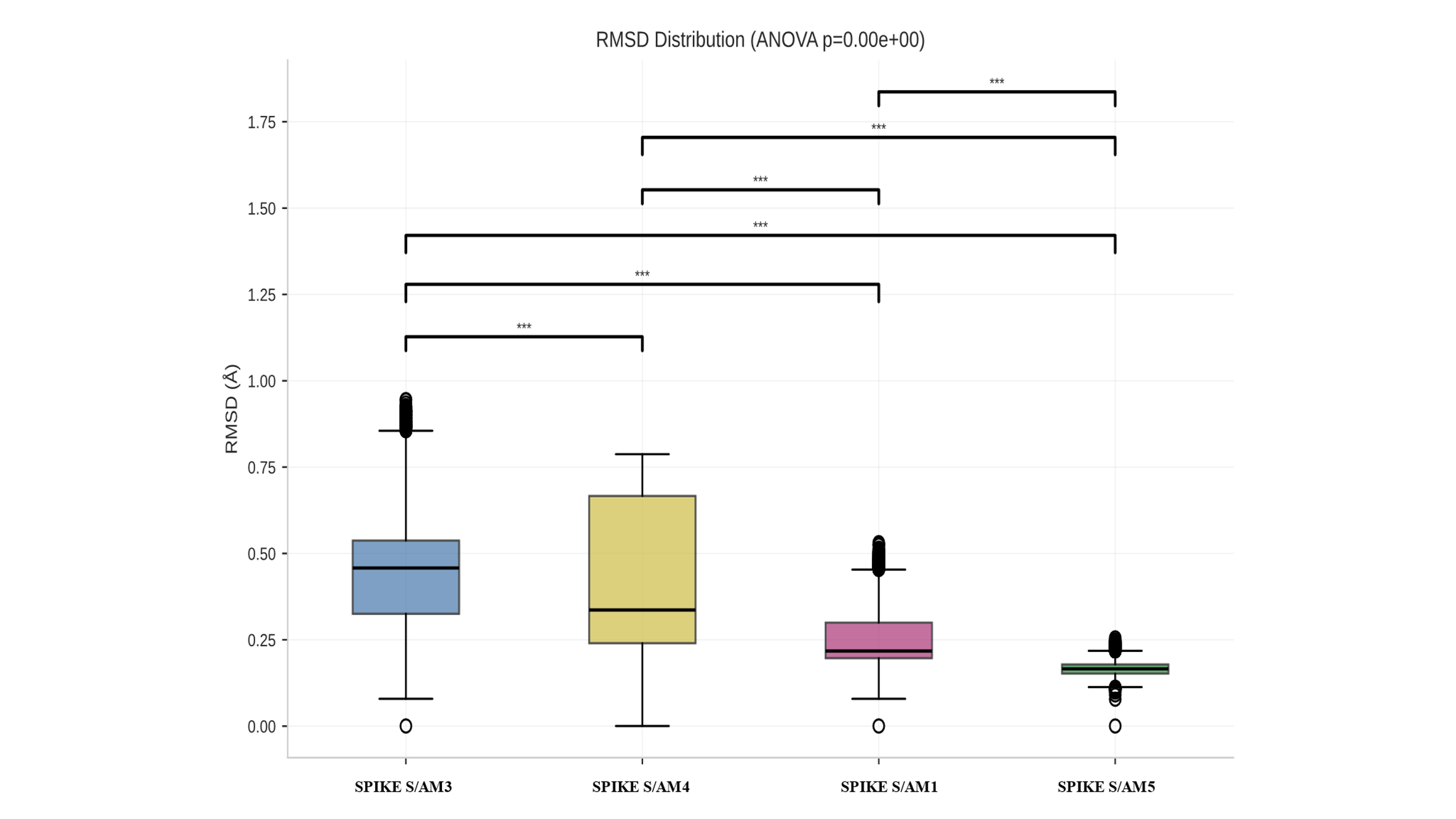


**Figure S1.** Distribution of root mean square deviations (RMSD) of Spike/peptide complexes, showing significant differences (ANOVA, p < 0.001)

*
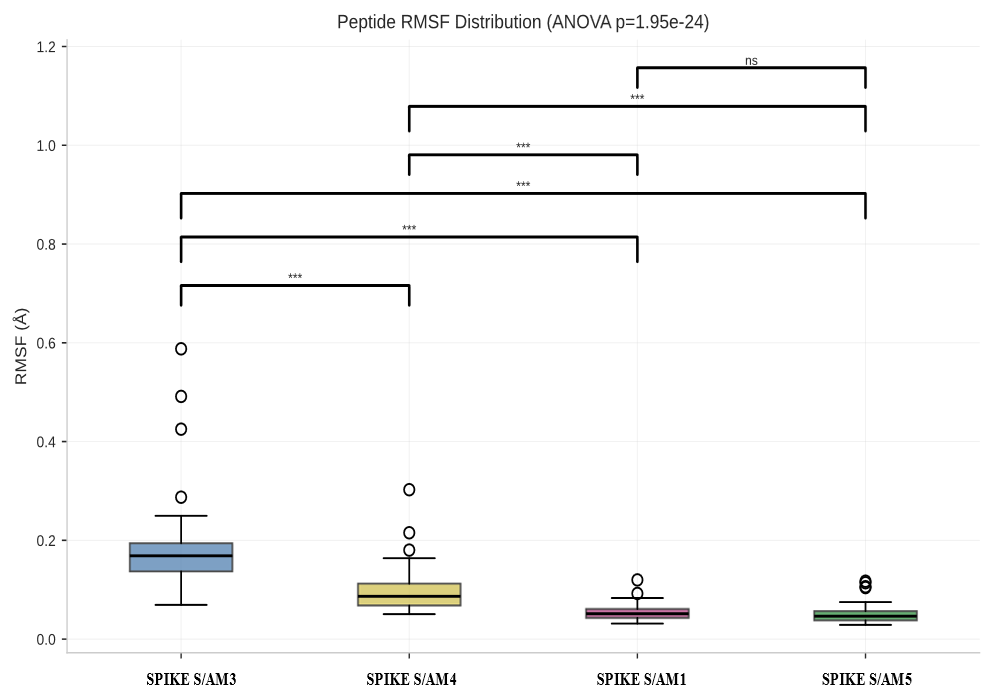
*

**Figure S2.** Distribution of root mean square fluctuations (RMSF) of Spike/peptide complexes, showing significant differences (ANOVA, p < 0.001)


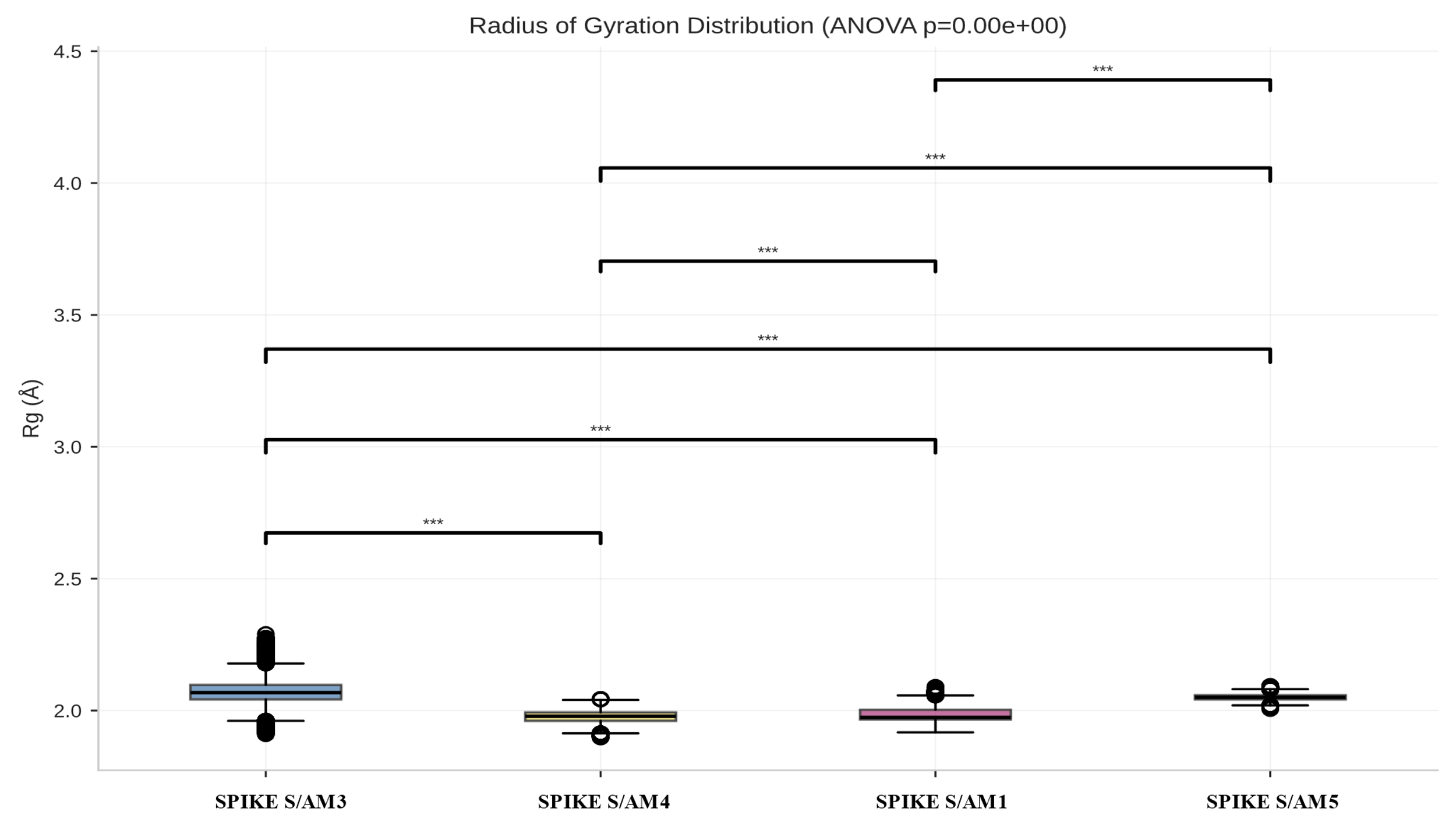


**Figure S3.** Distribution of radius of gyration distribution (Rg) of Spike/peptide complexes, showing significant differences (ANOVA, p < 0.001)


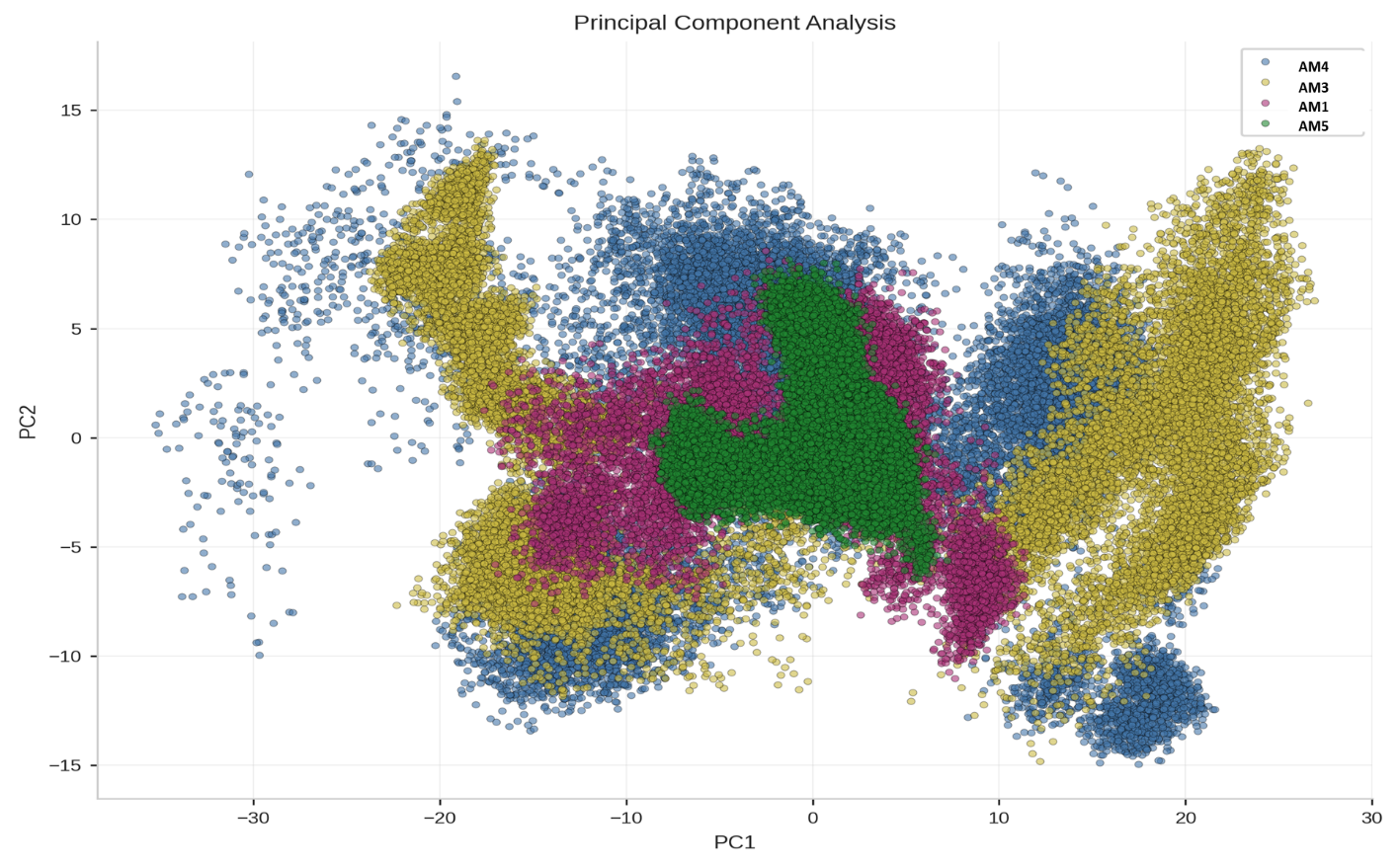


**Figure S4.** Principal Component Analysis reveals distinct conformational sampling of Spike S–peptide complexes.

Principal component analysis was performed on 200 ns molecular dynamics trajectories of Spike S protein in complex with peptides AM1 (violet), AM3 (yellow), AM4 (blue), and AM5 (green) from *Androctonus Mauretanicus* venom. Each point represents a conformational snapshot (sampled every 20 ps) projected onto the first two principal components (PC1 and PC2), which collectively account for 65% of the total variance (PC1: 42%, PC2: 23%). The clear separation of AM5 from other peptide complexes along PC1 demonstrates its unique ability to constrain Spike dynamics, consistent with superior binding affinity and inhibitory potential.
